# Human antibodies activate complement against *Plasmodium falciparum* sporozoites, and are associated with protection against malaria in children

**DOI:** 10.1186/s12916-018-1054-2

**Published:** 2018-04-30

**Authors:** Liriye Kurtovic, Marije C. Behet, Gaoqian Feng, Linda Reiling, Kiprotich Chelimo, Arlene E. Dent, Ivo Mueller, James W. Kazura, Robert W. Sauerwein, Freya J. I. Fowkes, James G. Beeson

**Affiliations:** 1 Burnet Institute, Melbourne, Australia; 2 Department of Immunology and Pathology, Monash University, Melbourne, Australia; 3 Department of Medical Microbiology, Radboud University Medical Center, Nijmegen, The Netherlands; 4 Kenya Medical Research Institute, Kisumu, Kenya; 5 Center for Global Health and Diseases, Case Western University, Cleveland, USA; 6 Division of Population Health and Immunity, Walter and Eliza Hall Institute, Melbourne, Australia; 7 Department of Parasites and Insect Vectors, Institut Pasteur, Paris, France; 8 Department of Epidemiology and Preventative Medicine and Department of Infectious Diseases, Monash University, Melbourne, Australia; 9 Centre for Epidemiology and Biostatistics, Melbourne School of Population and Global Health, The University of Melbourne, Melbourne, Australia; 10 Department of Microbiology, Monash University, Clayton, Australia; 11 Department of Medicine, The University of Melbourne, 185 Commercial Road, Parkville, Australia

**Keywords:** Antibodies, Circumsporozoite protein, Sporozoite, Complement, Malaria, *Plasmodium falciparum*, Vaccines

## Abstract

**Background:**

Antibodies targeting *Plasmodium falciparum* sporozoites play a key role in human immunity to malaria. However, antibody mechanisms that neutralize sporozoites are poorly understood. This has been a major constraint in developing highly efficacious vaccines, as we lack strong correlates of protective immunity.

**Methods:**

We quantified the ability of human antibodies from malaria-exposed populations to interact with human complement, examined the functional effects of complement activity against *P. falciparum* sporozoites *in vitro*, and identified targets of functional antibodies. In children and adults from malaria-endemic regions, we determined the acquisition of complement-fixing antibodies to sporozoites and their relationship with antibody isotypes and subclasses. We also investigated associations with protective immunity in a longitudinal cohort of children (*n* = 206) residing in a malaria-endemic region.

**Results:**

We found that antibodies to the major sporozoite surface antigen, circumsporozoite protein (CSP), were predominately IgG1, IgG3, and IgM, and could interact with complement through recruitment of C1q and activation of the classical pathway. The central repeat region of CSP, included in leading vaccines, was a key target of complement-fixing antibodies. We show that antibodies activate human complement on *P. falciparum* sporozoites, which consequently inhibited hepatocyte cell traversal that is essential for establishing liver-stage infection, and led to sporozoite death *in vitro*. The natural acquisition of complement-fixing antibodies in malaria-exposed populations was age-dependent, and was acquired more slowly to sporozoite antigens than to merozoite antigens. In a longitudinal cohort of children, high levels of complement-fixing antibodies were significantly associated with protection against clinical malaria.

**Conclusions:**

These novel findings point to complement activation by antibodies as an important mechanism of anti-sporozoite human immunity, thereby enabling new strategies for developing highly efficacious malaria vaccines. We also present evidence that complement-fixing antibodies may be a valuable correlate of protective immunity in humans.

**Electronic supplementary material:**

The online version of this article (10.1186/s12916-018-1054-2) contains supplementary material, which is available to authorized users.

## Background

Malaria is a substantial cause of global morbidity and mortality, and the effectiveness of current interventions is under threat due to increasing reports of drug and insecticide resistance. To achieve malaria control and elimination, there is a pressing need to develop and license highly efficacious malaria vaccines. This is a global priority, and the World Health Organization has set the ambitious goal to license a malaria vaccine that is at least 75% efficacious against clinical malaria by 2030 [1]. Currently, there is no licensed vaccine against *Plasmodium falciparum*, the main cause of malaria morbidity and mortality [2]. Vaccine development is significantly challenged by the lack of knowledge on the targets and mechanisms of functional immunity that confer protection against malaria, and a lack of correlates of protection. Defining these mechanisms is crucial for identifying strong correlates of protection, which will aid vaccine evaluation and enhance current vaccine candidates by inducing potent functional immune responses [3].

*Plasmodium* sporozoites are transmitted to humans when an infected female *Anopheles* mosquito probes the skin in search of a blood vessel [4]. Sporozoites are passively carried in the blood circulation to the liver, where they develop within hepatic cells, generating thousands of merozoites [5], which initiate blood-stage replication and subsequently cause symptomatic and severe malaria. Vaccines targeting sporozoites are attractive because fewer than 100 sporozoites are transmitted to humans [6] and blocking sporozoites will prevent infection and subsequent disease. Even partial immunity to sporozoites could reduce the number of successful hepatocyte infections and subsequent blood-stage parasitemia, which may confer protection against clinical malaria or reduce disease severity.

The predominant sporozoite surface antigen is circumsporozoite protein (CSP). Antibodies to CSP have been directly shown to confer protection against infection in animal malaria models [7, 8], have had some association with protection in human vaccine trials [9], and can be acquired through natural exposure to malaria [10, 11]. However, an enduring question that remains unanswered is how antibodies to sporozoites and CSP function to protect against infection. The most advanced malaria vaccine candidate, RTS,S/AS01 (RTS,S), is based on the central-repeat and C-terminal regions of CSP [12], and induces antibody and CD4+ T cell responses [13]. RTS,S was only partially efficacious against clinical malaria in phase III clinical trials (vaccine efficacy 29–36% in children aged 5–17 months after 48 months of follow-up) [14], and the functional mechanisms of vaccine-induced immunity remain unknown. These knowledge gaps severely impair our ability to enhance current vaccines and to design new vaccines that induce potent functional immunity for greater efficacy.

Sporozoites can take several hours to reach the liver [4], leaving them highly susceptible to immune attack at the inoculation site in the skin and in the circulation. The success of invasion and development in the liver is dependent on sporozoite motility, which includes: (i) gliding to migrate out of the skin [15], (ii) traversal to pass through cells, and (iii) hepatocyte invasion and maturation [16]. Anti-CSP antibodies can neutralize motility *in vitro*, although the relationship between function and protective immunity has not been demonstrated [17–19], and direct inhibitory activity of antibodies generally requires relatively high antibody concentrations [18, 20]. Furthermore, there are few data on whether the immunoglobulin G (IgG) subclass influences antibody function and what epitopes are primarily targeted.

We hypothesized that antibodies of the right type and specificity may fix and activate complement to neutralize *P. falciparum* sporozoites. Harnessing complement activity using antibodies is an attractive mechanism of functional immunity because complement could act early following inoculation to combat sporozoites (Additional file 1: Figure S1). Complement activation can occur via the classical pathway whereby C1q interacts with immune complexes, referred to as C1q-fixation [21]. This leads to sequential activation of downstream complement proteins such as C3, and formation of the membrane attack complex (MAC), which mediates cell death. Activation can also occur via the alternative pathway involving direct C3 activation against pathogens [21]. Currently it is unknown if *P. falciparum* sporozoites are susceptible to complement deposition and attack, and whether antibodies function to promote complement activation and consequently neutralize sporozoite function or lead to sporozoite death.

We investigated the ability of antibodies to CSP and *P. falciparum* sporozoites to recruit human complement proteins, and demonstrated the functional effects of complement activation against sporozoites *in vitro*. We also explored the natural acquisition of complement-fixing antibodies, and then assessed whether functional antibodies were associated with protection in a prospective longitudinal cohort of children.

## Methods

### Aims and study participants

There were several aims to this study: (i) to determine whether human antibodies fix complement, (ii) to explore the relationship between functional antibodies and isotype, subclass, and specificity, (iii) to examine the natural acquisition of functional antibodies in young children, and (iv) to assess the association between functional antibodies and protection against malaria in children. To meet these aims, we tested antibody samples from previously described cohort studies as summarized below. Note that all three study sites were considered to be malaria holo- or hyper-endemic, and no bed nets of any type were in place at this time.

(i) To maximize the likelihood of detecting functional anti-CSP antibodies, we tested two cohorts of malaria-exposed adults from distinct areas, as anti-CSP antibodies have been previously reported to be acquired with age as a result of repeated malaria exposure. Sera from adults living in Madang District (Papua New Guinea, PNG, *N* = 116) were collected in 2001 and 2002 [22], and plasma from adults living in the Kanyawegi subdistrict, Kisumu county (Kenya, *N* = 104) were collected in August 2007 [23].

(ii) We then preferentially selected 30 samples from each adult cohort (PNG and Kenyan) to characterize the antibody response further, based on IgG/C1q-fixation reactivity to CSP and sample availability.

(iii) The majority of malaria morbidity occurs in children under 5 years, and so we tested a cohort of young children (0.3–5.9 years) to examine the acquisition of functional antibodies, and compared this cohort to adults from the same area. Plasma from children/adults living in the Chulaimbo subdistrict, Kisumu county (Kenya, *N* = 75) were collected in February and March 2007 [24]. Samples were categorized into groups based on the median ages of 0.5, 1, 2, 5 and 35 years (age ranges were 0.3–0.7, 1–1.5, 1.8–3.0, 4.7–5.9, and 19.6–69.2 with *n* = 11, *n* = 14, *n* = 18, *n* = 16, and *n* = 16, respectively).

(iv) To assess the relationship between functional antibodies and protection against malaria, we tested samples from a longitudinal prospective treatment-to-reinfection study. Plasma samples from children living in Madang Province (PNG, *N* = 206) aged 5–14 years (median = 9.3 years) were collected at enrolment in 2004 and 2005 [25]. Children were treated to clear parasitemia present at baseline, and then actively followed every 2 weeks for symptomatic illness and parasitemia, and by passive case detection over a period of 6 months. A clinical episode of *P. falciparum* malaria was defined as fever and *P. falciparum* parasitemia >5000 parasites/μl.

We also generated two pools of Kenyan donors with high C1q-fixation reactivity from the adult Kenyan cohort. Pool 1 (*n* = 10) was used in IgG-purification experiments, using the Melon gel purification kit as described by the manufacturer (Thermo Fisher Scientific, Waltham, USA). Pool 2 (*n* = 19) was used in the *in vitro* hepatocyte traversal experiments. To minimize potential background reactivity from any residual complement that may remain after heat inactivation, antibodies from pool 2 (and a control pool from residents of Melbourne, Australia) were purified by ammonium sulfate precipitation [26]. Sterile saturated ammonium sulfate was slowly added with continuous agitation to plasma (diluted 1/5 in sterile saline) to a final concentration of 50%. Following 30 min incubation on ice, the precipitate was pelleted (20,000 relative centrifugal force (RCF) × 10 min), then washed in 50% sterile saturated ammonium sulfate. The precipitate was repeatedly washed in excess phosphate-buffered saline (PBS) using a centrifugation concentration device, as described by the manufacturer (100 kDa cut-off, Millipore, Burlington, USA), and re-suspended to the same starting volume of plasma.

### Parasites

Aseptic, purified, non-attenuated cryopreserved *P. falciparum* 3D7 sporozoites (Sanaria, Rockville, USA) were provided by PATH’s Malaria Vaccine Initiative or purchased from Sanaria. Freshly dissected *P. falciparum* NF54 and NF166.C8 sporozoites were generated at Radboud University Medical Center (Radboudumc, Nijmegen, The Netherlands), as described previously [20, 27].

### Antigens and antibodies

Recombinant *P. falciparum* CSP was expressed in *Pichia pastoris* and purified using high-performance liquid chromatography (Sanaria) [28]. The protein was based on the 3D7 sequence beginning at amino acid residue 50 and contained 22 NANP repeats and four NVDP repeats. A peptide representative of the *P. falciparum* CSP central repeat B-cell epitope (NANP)_15_ was synthesized and purified using high-performance liquid chromatography (Life Tein, Hillsborough, USA). Full-length recombinant merozoite surface protein 2 (MSP2) was expressed in *Escherichia coli* as previously described [29].

Rabbits were immunized with three doses of 200 μg CSP 4 weeks apart (the first immunization was administered in complete Freud’s adjuvant and the last two in incomplete Freud’s adjuvant). The terminal bleed was obtained 12 days after the final immunization, and anti-CSP IgG was purified at the Walter and Eliza Hall Institute. Pre-immune IgG was purified in-house using the Melon gel purification kit, as described by the manufacturer (Thermo Fisher Scientific). Animal immunizations were approved by the Animal Ethics Committee of the Walter and Eliza Hall Institute. We were provided with subclass switched mouse anti-NANP monoclonal antibodies (MAbs) 2H8-IgG1/IgG2a and 3C1-IgG1/IgG3 (LakePharma, Belmont, USA) by PATH’s MVI [30].

### Antibody isotypes and subclasses by enzyme-linked immunosorbent assay

The procedure for the enzyme-linked immunosorbent assay (ELISA) was as follows. Flat bottom 96-well MaxiSorp plates (Thermo Fisher Scientific) were coated with 0.5 μg/ml antigen in PBS overnight at 4 °C. Plates were washed thrice in PBS-Tween20 0.05% (v/v), blocked using 1% (w/v) casein in PBS for 2 h at 37 °C, and then washed again. Human antibody samples were applied in duplicate at 1/2000 (IgG/M) or 1/500 (IgG subclasses) dilutions in 0.1% (w/v) casein in PBS (diluting buffer) for 2 h at room temperature (RT), and then washed. Antibody isotypes were detected using goat anti-human IgG/IgM horse radish peroxidase (HRP) conjugated antibodies (Millipore) at 1/2500 dilution for 1 h at RT, followed by washing and incubation with 2,2′-azino-bis(3-ethylbenzothiazoline-6-sulfonic acid) (ABTS) substrate (Sigma-Aldrich, St. Louis, USA) for 1 h at RT in the dark. The reaction was stopped using 1% (w/v) sodium dodecyl sulfate in PBS. The optical density (OD) was measured at 405 nm. IgG subclasses were detected using mouse anti-human IgG1/IgG2/IgG3/IgG4 antibodies (Invitrogen) at 1/1000 dilution for 1 h at RT, followed by washing and incubation with goat anti-mouse IgG HRP conjugated antibodies (Millipore) at 1/1000 dilution for 1 h at RT. Plates were washed and incubated with a tetramethylbenzidine substrate (Sigma-Aldrich) for 1 h at RT in the dark, and the reaction was stopped using 1 M sulfuric acid (OD measured at 450 nm). Rabbit and murine antibodies were detected using goat anti-rabbit/mouse HRP conjugated antibodies (Millipore, Abcam) at 1/2000 and ABTS substrate.

### Complement-fixation by ELISA and Western blot

Again, 96-well flat bottom MaxiSorp plates were coated with antigen, blocked and washed as described above. Human antibody samples were applied in duplicate at 1/100–1/250 dilution for 2 h at RT, and then washed. Plates were incubated with 10 μg/ml human C1q, C1q-depleted serum (Millipore), C5-depleted serum (for C2/C3/C4 fixation, Millipore) or fresh pooled non-immune serum (for C5b-C9 fixation) at 1/10 dilution for 30 min at RT, washed, and detected using anti-C1q/C2/C3/C4/C5b-C9 antibodies at 1/1000–1/2000 dilution (Dako, Santa Clara, USA; Abcam, Cambridge, UK; Millipore) for 1 h at RT. Plates were washed and incubated with the appropriate HRP conjugated antibodies (Abcam, Sigma-Aldrich, Millipore) at 1/2000 dilution for 1 h at RT followed by washing. Complement-fixation was detected using a tetramethylbenzidine substrate as described above (MSP2 experiments utilized ABTS). Complement-fixation using murine and rabbit antibodies were conducted using the same methods.

Complement-fixation to 3D7 *P. falciparum* sporozoites by ELISA was conducted using the same method as above (coating of 10,000 cells per well); however, all incubation times did not exceed 1 h, were conducted at RT, and the washing buffer contained PBS only. To measure complement-fixation by Western blot, 10,000 sporozoites were incubated with human antibody samples at 1/10 dilution and fresh normal human serum at 25% in PBS for 15 min at 37 °C with agitation. Sporozoites were washed twice in cold protease inhibitor (1600 x RCF, 4 min), processed for SDS-PAGE and then blotted into nitrocellulose membrane (under reducing conditions). The membrane was blocked in 10% (w/v) skimmed milk in PBS-Tween20 0.5% (v/v) and probed using anti-C1q/iC3b antibodies (Serotec, Kidlington, UK) followed by HRP conjugated antibodies, with membrane stripping (Sigma-Aldrich), washing and blocking in between. Protein bands were detected using chemiluminescent substrate and autoradiography film (GE Life Sciences, Chicago, USA). Membranes were also probed with anti-CSP antibodies as a loading control.

### Experimental controls and standardization

Human antibody samples (serum/plasma) tested by ELISA were excluded if duplicates varied by greater than 25% (unless duplicates differed by OD < 0.1). OD values were corrected by subtracting the background reactivity of control wells containing no serum or plasma. Cohort experiments that spread across multiple ELISA plates were standardized for plate-to-plate variation using positive control wells containing highly reactive samples from malaria-exposed individuals. Malaria-naïve sera from Melbourne donors were tested as negative controls. Test samples giving ODs greater than the mean + 3 standard deviations of malaria naïve controls were considered positive. This positive cut-off value was used to convert OD into arbitrary units (AU) as follows: AU = sample corrected OD / positive cut-off OD, so that samples with AU > 1 were considered positive. All plasma/serum samples were heat inactivated at 56 °C for 45 min prior to use.

### Sporozoite cell traversal

Traversal was conducted as previously described [20] using freshly dissected *P. falciparum* NF54 and NF166.C8 sporozoites. Briefly, sporozoites were pre-incubated with pooled human antibody samples (malaria-exposed Kenyan and malaria-naïve Melbourne pools purified by ammonium sulfate precipitation) at 1/10 dilution, or vaccine-induced rabbit anti-CSP IgG and pre-immune IgG at 1 and 10 μg/ml, for 30 min on ice. Sporozoite/antibody samples were added to HC-04 cells seeded on 96-well plates, along with 10% normal human serum (NHS; active complement) or heat-inactivated serum (HIS; inactive complement), and tetramethylrhodamine (Rh) labelled dextran (50,000 sporozoites and 50,000 HC-04 cells per well). Sporozoites were allowed to traverse HC-04 cells under these conditions (in the presence of immune/non-immune antibodies and active/inactive complement) for 2 h at 37 °C in 5% CO_2_, and were then washed in PBS. HC-04 cells were then trypsinized, washed in 10% fetal bovine serum in PBS, and re-suspended in 1% paraformaldehyde. Traversed HC-04 cells were measured as Rh positive, and quantified by flow cytometry using the Cyan (Beckman Coulter) and FlowJo software (gating strategy shown in Additional file 1: Figure S2A). The percentage of Rh positive cells was corrected for background reactivity using HC-04 cells treated with dextran only. Traversal inhibition by immune antibodies (Kenyan pool and rabbit anti-CSP IgG) compared to non-immune antibodies (Melbourne pool and rabbit pre-immune IgG) in the presence of NHS and HIS was calculated as

$$ \left(1-\frac{\%\mathrm{traversed}\ \mathrm{cells}\ \mathrm{in}\ \mathrm{the}\ \mathrm{presence}\ \mathrm{of}\ \mathrm{immune}\ \mathrm{antibodies}}{\%\mathrm{traversed}\ \mathrm{cells}\ \mathrm{in}\ \mathrm{the}\ \mathrm{presence}\ \mathrm{of}\ \mathrm{non}\hbox{-} \mathrm{immune}\ \mathrm{antibodies}}\right)\times 100 $$

Sporozoites were also pre-incubated with anti-CSP MAb 3SP2 as a positive control.

### Sporozoite cell death by flow cytometry

Altogether, 50,000 3D7 *P. falciparum* sporozoites were incubated with rabbit anti-CSP antibodies at 1/10 dilution and 20% human serum (normal or C5-depleted) for 10 min at 37 °C. Sporozoites were placed on ice and dead cells were stained using the DNA-binding dye, propidium iodide (PI). PI positive cells were quantified by flow cytometry using FACSVerse (BD Biosciences) and FlowJo software (gating strategy shown in Additional file 1: Figure S2B).

### Statistical analysis

Data were analyzed using GraphPad Prism version 6.05 and Stata 13.1. The following two-tailed non-parametric tests were performed where appropriate: Spearman’s correlation coefficient (rho), Wilcoxon matched-pairs signed rank test, Man Whitney U test, and Kruskal–Wallis test. To investigate the association between anti-CSP IgG and IgM with C1q fixation in PNG adults, we performed a multiple linear regression with robust standard errors. To evaluate associations between specific antibodies (C1q fixation activity or IgG reactivity to CSP) and malaria risk in the longitudinal study, Kaplan–Meier survival curves were plotted to show the cumulative proportion of children who had experienced an episode of malaria during follow-up, stratified by antibody response group defined as negative, low positive, and high positive (positive samples were defined as those with reactivity > 3 standard deviations above the mean of the malaria-naïve control samples, high-positive samples were those defined as being above the median of the positive samples, and low positive were those below the median of positive samples). Hazard ratios (HRs) of the risk of malaria during follow-up were calculated using the Cox proportional hazards model (using the first episodes of malaria only), and adjusted HRs were calculated by including age, location of residence, and parasitemia status at enrolment [25].

## Results

### Naturally acquired anti-CSP antibodies are predominately IgG1, IgG3, and IgM

**Fig. 1:**
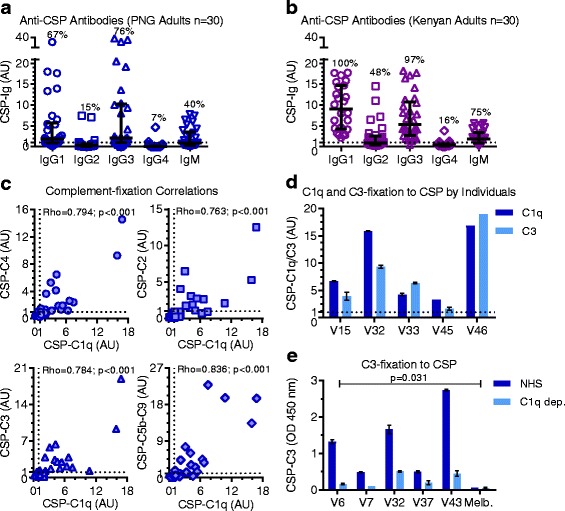
Naturally acquired human anti-CSP antibodies are predominately IgG1, IgG3, and IgM, and can promote complement fixation to CSP. Antibodies from malaria-exposed adults (*n* = 30 in each group) living in PNG (**a**, **c-e**) and Kenyan (**b**) were tested for IgG/IgM and complement-fixation to CSP by ELISA. Results were standardized to arbitrary units (AU) based on malaria-naïve negative controls from Melbourne (seropositivity defined as AU > 1, shown as dotted lines), and mean and range of duplicates were graphed (mean only for scatter plots). **a,b** IgG subclasses and IgM reactivity to CSP. The median, interquartile range, and percentage of positive samples are shown. **c** Correlations between C1q-fixation and C4/C3/C3/C5b-C9-fixation to CSP (Spearman’s correlation coefficient, rho). **d** Examples of C1q and C3-fixation to CSP by individual serum samples (V15, V32, V33, V45, and V46 from PNG donors, *n* = 5). **e** C3-fixation to CSP by individual samples (V6, V7, V32, V37, and V43 from PNG donors, *n* = 5) in the presence of normal human serum (NHS) and serum depleted of C1q (C1q dep.) (Wilcoxon matched-pairs signed rank test). Non-standardized data are shown, as only one Melbourne control (Melb.) was tested. AU arbitrary units, CSP circumsporozoite protein, dep. depleted, ELISA enzyme-linked immunosorbent assay, Melb. Melbourne, NHS normal human serum, PNG Papua New Guinea

Antibody isotype and subclass influence the ability to fix C1q and activate the classical pathway. Therefore, we characterized naturally acquired antibodies to the most abundant sporozoite surface protein, CSP, for possible interaction with complement. We measured antibody levels by ELISA and converted OD into AU. Among a selection of malaria-exposed adults from PNG and Kenya (*n* = 30 in each group), 40% and 75% were positive for anti-CSP IgM, respectively (Fig. 1a and b). IgM antibodies are potent complement activators whereas IgG complement activity is mediated primarily by the IgG1 and IgG3 subclasses, whereby IgG3 generally has greater activity [31]. The predominant subclasses in PNG and Kenyan adults were the complement-fixing subclasses IgG1 (seropositivity 67% and 100%, respectively) and IgG3 (seropositivity 76% and 97%, respectively). In contrast, the seroprevalence of IgG2 and IgG4 antibodies were lower (PNG seropositivity 15% and 7%; Kenyan seropositivity 48% and 16%, respectively).

### Naturally acquired human antibodies and vaccine-induced antibodies promote complement-fixation to recombinant CSP

**Fig. 2:**
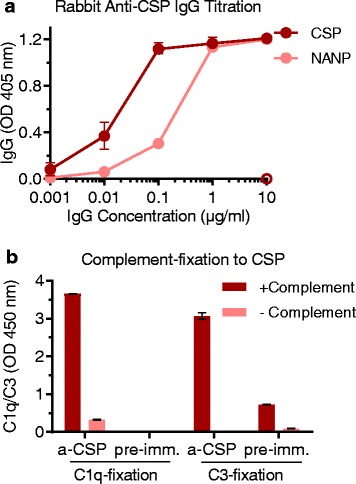
Vaccine-induced rabbit anti-CSP IgG can fix human complement proteins to CSP. Purified IgG from rabbit serum before (pre-imm.) and after (a-CSP) CSP immunization was tested for IgG and complement-fixation to CSP by ELISA. Results were corrected for background reactivity using no-IgG controls, and the mean and range of the duplicates were graphed. **a** IgG reactivity to CSP and (NANP)_15_ peptide (pre-immune IgG shown with the open symbol was tested at 10 μg/ml). Results from two independent experiments are shown. **b** C1q and C3-fixation to CSP tested in the presence (+) and absence (−) of complement to confirm specificity for complement fixation. a-CSP after CSP immunization, CSP circumsporozoite protein, ELISA enzyme-linked immunosorbent assay, IgG immunoglobulin G, pre-imm. before CSP immunization

We next investigated whether naturally acquired antibodies in humans and vaccine-induced antibodies in animals were capable of promoting complement activation. Since the classical pathway is initiated by antibody-C1q interactions [31, 32], we tested whether antibodies to CSP could fix C1q using ELISA-based methods. Among a selection of PNG adults (*n* = 30), 77% of individuals were positive for this activity. Furthermore, human anti-CSP antibodies also fixed other complement components downstream of C1q, including C4, C2, C3, and C5b-C9 (reflecting MAC-formation) [21], all of which strongly positively correlated with C1q-fixation (Spearman’s correlation coefficient rho = 0.794, 95% confidence interval (CI) 0.591 to 0.903; rho = 0.763, 95% CI 0.548 to 0.884; rho = 0.784, 95% CI 0.584 to 0.895; and rho = 0.836, 95% CI 0.674 to 0.921; *p* < 0.001 for all tests, respectively) (Fig. 1c and d). We confirmed that C3-fixation was mediated by anti-CSP antibodies and the classical pathway and not by the alternative pathway using C1q-depleted serum as a source of complement, which significantly reduced reactivity by 61–88% (*p* = 0.031) (Fig. 1e). We next addressed whether complement-fixing antibodies could be elicited by CSP immunization of rabbits, as rabbit IgG is known to engage [33] and activate the human complement [34]. Vaccine-induced IgG demonstrated strong reactivity to recombinant CSP and to the central repeat region of CSP represented by the synthetic (NANP)_15_ peptide, and promoted C1q and C3-fixation to CSP (Fig. 2). Collectively these data demonstrate that antibodies can fix C1q and activate the classical pathway against CSP.

### Anti-CSP antibody isotype and subclass are associated with complement-fixation

**Fig. 3:**
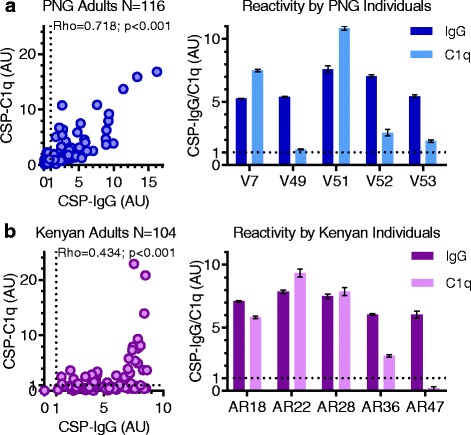
Naturally acquired human anti-CSP IgG correlates with the ability to promote C1q-fixation to CSP, despite individual differences in reactivity. Antibodies from malaria-exposed adults living in PNG (**a**; *N* = 116) and Kenya (**b**; *N* = 104) were tested for IgG and C1q-fixation to CSP by ELISA. Results were standardized to arbitrary units (AU) based on malaria-naïve negative controls from Melbourne (seropositivity defined as AU > 1, shown as dotted lines), and the mean and range of duplicates were graphed (mean only for scatter plots). The left panels show correlations between IgG and C1q-fixation to CSP (Spearman’s correlation coefficient, rho). The right panels show selected examples of IgG and C1q-fixation to CSP for individual serum samples from PNG donors (V7, V49, V51, V52, and V53) and Kenyan donors (AR18, AR22, AR28, AR36, and AR47). AU arbitrary units, CSP circumsporozoite protein, ELISA enzyme-linked immunosorbent assay, IgG immunoglobulin G, PNG Papua New Guinea

We examined the relationship between IgG and C1q-fixation to CSP in two different cohorts of malaria-exposed adults from PNG (*n* = 116) and Kenya (*n* = 104). Although there was a consistent positive trend between IgG and C1q-fixation in both populations, we observed marked differences in the ability to fix C1q among individuals with similar levels of anti-CSP IgG (Fig. 3). This suggests that additional factors or antibody properties influence this function.

To confirm that IgG alone is capable of promoting C1q-fixation, we compared purified IgG and whole plasma from a pool of Kenyan adults with high levels of C1q-fixing antibodies (*n* = 10). Purified IgG could fix C1q, but less effectively than plasma, possibly due to the loss of IgM, which is known to activate complement potently (Additional file 1: Figure S3A). To explore in more detail the relative contributions of IgG and IgM in promoting C1q-fixation, we performed a linear regression with data from PNG adults (*n* = 116). For every unit increase in anti-CSP IgG, there was an increase in C1q-fixation of 0.785 AU (95% CI 0.585 to 0.985; *p* < 0.001), and this value was lower after adjusting for anti-CSP IgM (0.579, 95% CI 0.342 to 0.851; *p* < 0.001) (Additional file 1: Table S1). The inclusion of IgM only slightly improved the fit of the model (IgG R-squared = 0.67 and IgG/IgM R-squared = 0.71), suggesting that most variation in C1q-fixation responses could be explained by anti-CSP IgG for these samples.

Of the IgG subclasses, IgG1 and IgG3 are the most potent at activating the classical complement pathway. As expected, anti-CSP IgG1 and IgG3 both strongly correlated with C1q-fixation to CSP among a selection of PNG adults (*n* = 30, rho = 0.829, 95% CI 0.662 to 0.917 and rho = 0.792, 95% CI 0.592 to 0.900; *p* < 0.001 for both tests). These antibodies also strongly correlated with C4, C2, C3, and C5b-C9-fixation (Table 1), which further supported that complement activation was via the antibody-dependent classical pathway. To identify which subclass was prevalent in samples with high levels of C1q-fixing antibodies (above median AU = 2.2, *n* = 14), we compared the ratio of anti-CSP IgG1 to IgG3 (Additional file 1: Figure S3B). There tended to be an increased IgG3 response, whereas the ratio of anti-CSP IgG to IgM was highly variable, indicating that IgM may be important in some individuals (Additional file 1: Figure S3C). Overall, characterizing the antibody response is an important consideration when assessing functional antibody responses.

**Table 1:** Correlations between antibodies and complement fixation to CSP among malaria-exposed PNG adults (*n* = 30)

Antibody	Rho C1q	Rho C4	Rho C2	Rho C3	Rho C5b-C9
IgG	0.831 (0.666 to 0.919)	0.813 (0.625 to 0.912)	0.797 (0.606 to 0.901)	0.831 (0.666 to 0.919)	0.803 (0.616 to 0.904)
IgG1	0.829 (0.662 to 0.917)	0.839 (0.672 to 0.925)	0.830 (0.664 to 0.918)	0.895 (0.784 to 0.950)	0.745 (0.518 to 0.874)
IgG2	0.607 (0.276 to 0.809)	0.443 (0.046 to 0.720)	0.404 (0.008 to 0.691)	0.552 (0.198 to 0.779)	0.485 (0.108 to 0.740)
IgG3	0.792 (0.592 to 0.900)	0.795 (0.586 to 0.904)	0.788 (0.585 to 0.898)	0.801 (0.608 to 0.905)	0.754 (0.527 to 0.880)
IgG4	0.416 (0.039 to 0.689)	–	–	–	–
IgM	0.826 (0.656 to 0.916)	0.655 (0.363 to 0.830)	0.620 (0.325 to 0.805)	0.738 (0.506 to 0.870)	0.791 (0.596 to 0.898)

Spearman’s correlation coefficient (rho) and 95% CI between anti-CSP antibodies and complement fixation to CSP. Values greater than 0.7 are underlined. Only significant correlations (*p* < 0.05) are shown

*CI* confidence interval, *CSP* circumsporozoite protein, *PNG* Papua New Guinea

### *P. falciparum* sporozoites are susceptible to complement-fixation by human antibodies

**Fig. 4:**
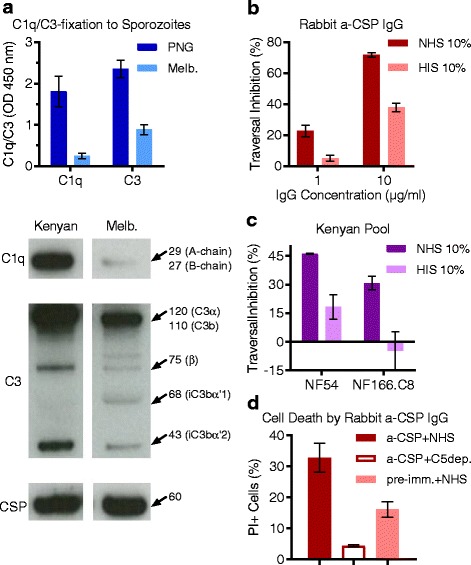
Antibodies promote complement-fixation on *P. falciparum* sporozoites, which enhances antibody-mediated traversal inhibition and can lead to sporozoite death. **a** Antibody samples from malaria-exposed (PNG and Kenyan) and malaria-naïve (Melbourne) individuals were tested for the ability to fix human C1q and C3 to 3D7 *P. falciparum* sporozoites by ELISA and Western blot. ELISA samples (top panel) were tested in duplicate, and the mean and range were graphed (C1q-fixation data is from two independent experiments). Western blot sporozoites (bottom panel) were incubated with human antibody samples and normal human serum (NHS, active complement), and then washed and processed for Western blotting under reduced conditions. Any complement proteins that had deposited on the sporozoite surface were detected using C1q- and C3-specific antibodies, and the sporozoite surface antigen, CSP, was used as a loading control. **b,c** *In vitro* traversal inhibition of freshly dissected sporozoites incubated with HC-04 cells, in the presence of NHS and heat-inactivated human serum (HIS). Each condition was tested in duplicate, and the mean and range were graphed. **b** Traversal inhibition of NF54 sporozoites treated with rabbit anti-CSP IgG (compared to rabbit pre-immune IgG). **c** Traversal inhibition of NF54 and NF166.C8 sporozoites treated with malaria-exposed Kenyan pool (compared to malaria-naïve Melbourne pool). Results from two independent experiments are shown. **d** Percentage of dead 3D7 sporozoites (PI+ cells) treated with rabbit anti-CSP IgG and pre-immune IgG, in the presence of NHS or C5-depleted serum (C5dep.). The mean and range of two independent experiments are graphed. a-CSP after CSP immunization, C5dep. C5-depleted serum, CSP circumsporozoite protein, ELISA enzyme-linked immunosorbent assay, HIS heat-inactivated human serum, IgG immunoglobulin G, Melb. Melbourne, NHS normal human serum, OD optical density, PI propidium iodide, PNG Papua New Guinea, pre-imm. before CSP immunization

Initially, we tested antibodies from a highly reactive PNG adult for the ability to promote complement-fixation on sporozoites by ELISA (Fig. 4a; reactivity to CSP shown in Additional file 1: Figure S4A). Consistent with complement-fixation to CSP, we observed greater C1q and C3-fixation in the presence of antibodies from the PNG individual than the malaria-naïve individual. It is noteworthy that some C3-fixation was observed in the presence of malaria-naïve antibodies, suggesting that C3 may bind directly to the sporozoite surface to some extent, although this was substantially lower than C3-fixation in the presence of malaria-exposed antibodies. To confirm these findings, we tested antibodies from a highly reactive Kenyan adult for complement-fixation on sporozoites by Western blot (Fig. 4a; reactivity to CSP shown in Additional file 1: Figure S4A). C1q and enhanced C3-fixation occurred in the presence of malaria-exposed antibodies, and some direct C3-fixation was also observed in the presence of malaria-naïve antibodies (repeat Western blots with reduced exposure time to observe the C3b band more clearly are shown in Additional file 1: Figure S4B). These data show that naturally acquired antibodies from malaria-exposed individuals promote complement fixation on sporozoites via the antibody-dependent classical pathway.

### Sporozoite traversal inhibition by human and vaccine-induced rabbit antibodies is enhanced by complement

We examined whether antibodies inhibited sporozoite traversal, and whether this was enhanced in the presence of an active complement. Sporozoites rely on their traversal form of motility to pass through the sinusoidal cell layer and several hepatic cells before encountering a terminal hepatocyte for cellular invasion and development [16]. We tested rabbit anti-CSP IgG previously shown to fix the human complement to evaluate whether the functional effect of antibodies to CSP was enhanced by complement. Rabbit anti-CSP IgG inhibited sporozoite traversal of HC-04 cells (compared to rabbit pre-immune IgG), and inhibitory activity was substantially enhanced in the presence of NHS compared to HIS, whereby complement proteins were active and inactive, respectively (Fig. 4b). To establish whether complement also enhanced the function of human antibodies that contain antibodies to CSP and other *Plasmodium* antigens, we tested purified antibodies (including IgG and IgM) from a pool of malaria-exposed Kenyan adults (*n* = 19 samples, Additional file 1: Figure S5) and malaria-naïve Melbourne donors, and quantified *P. falciparum* sporozoite transversal (Fig. 4c). Kenyan antibodies inhibited NF54 sporozoite traversal (compared to Melbourne antibodies) twofold higher in the presence of an active complement compared to an inactive complement (mean and range of two independent experiments: 46% [45.7 to 46.2%] and 18.2% [11.8 to 24.6%], respectively). We confirmed this effect using sporozoites from a second *P. falciparum* strain, NF166.C8, whereby inhibition of antibody-mediated traversal was observed only in the presence of active complement and was not detected when antibodies were tested with inactivated complement (30.7% [27.1 to 34.2%] and −4.7% [−14.6 to 5.2%], respectively). Therefore, the ability of antibodies to inhibit sporozoite traversal was enhanced in the presence of active human complement.

### Antibody-mediated complement-fixation leads to sporozoite death

We examined whether sporozoites were susceptible to complement-mediated death since the formation of the MAC can lead to membrane damage and possible lysis of target cells. Cell death caused by complement might be a contributing mechanism in the inhibition of cell traversal. *P. falciparum* sporozoites were incubated with rabbit anti-CSP IgG and NHS containing an active complement, or C5-depleted serum as a negative control as MAC-formation is C5 dependent (Fig. 4d). We observed a twofold increase in the percentage of dead cells (PI positive) [35] when sporozoites were incubated with complement and anti-CSP IgG compared to pre-immune IgG (mean and range of two independent experiments: 32.8% [28.1 to 37.4%] and 16.1% [13.6 to 18.6%], respectively). Few PI-positive cells were detected following treatment with anti-CSP IgG and C5-depleted serum (4.4% [4.1 to 4.7%]). These data show that anti-CSP IgG and complement proteins interact to enhance complement-mediated sporozoite death.

### The NANP-repeat epitope is a target of C1q-fixing antibodies

**Fig. 5:**
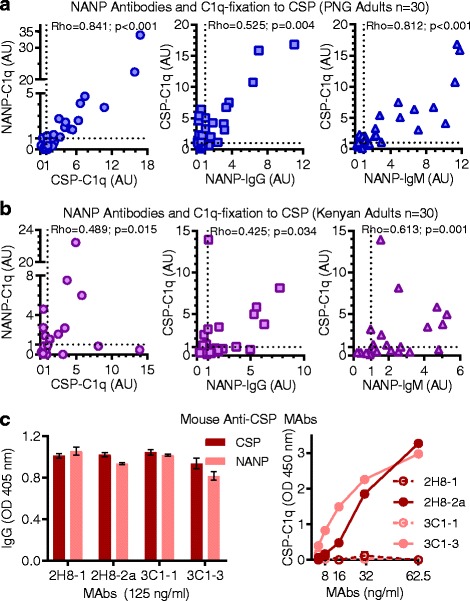
Antibodies that target the NANP epitope of CSP can promote C1q-fixation. Antibodies from malaria-exposed adults living in PNG (*n* = 30) (**a**) and Kenya (*n* = 30) (**b**) were tested for IgG, IgM, and C1q-fixation to (NANP)_15_ peptide by ELISA, and correlated with C1q fixation to CSP (Spearman’s correlation coefficient, rho). Results were standardized to arbitrary units (AU) based on malaria-naïve negative controls from Melbourne (seropositivity defined as AU > 1, shown as dotted lines) and mean of duplicates were graphed. **c** Mouse anti-CSP MAbs 2H8-IgG1/IgG2a and 3C1-IgG1/IgG3 were tested for CSP-IgG, NANP-IgG, and C1q-fixation to CSP by ELISA. Results were corrected for background reactivity using no-IgG controls, and the mean and range of duplicates were graphed. AU arbitrary units, CSP circumsporozoite protein, ELISA enzyme-linked immunosorbent assay, IgG immunoglobulin G, IgM immunoglobulin M, MAb monoclonal antibody, OD optical density, PNG Papua New Guinea

The NANP-repeat sequence is a major B-cell epitope of *P. falciparum* CSP and a key target of antibodies generated by the RTS,S vaccine [36]. Among PNG adults (*n* = 30), 55% were positive for C1q-fixation to (NANP)_15_ peptide and 77% were positive for C1q-fixation to CSP. These variables were strongly correlated (rho = 0.841, 95% CI 0.679 to 0.925; *p* < 0.001), suggesting that NANP was a significant target of C1q-fixation to CSP (Fig. 5a). Among Kenyan adults (*n* = 30), a lower proportion of individuals were positive for C1q-fixation to (NANP)_15_ and CSP (36% and 44%, respectively), and these variables only moderately correlated (rho = 0.489, 95% CI 0.094 to 0.751; *p* = 0.015) (Fig. 5b).

IgG and IgM isotype reactivity of PNG samples to (NANP)_15_ positively correlated with C1q-fixation to CSP (rho = 0.525, 95% CI 0.185 to 0.752; *p* = 0.004 and rho = 0.812, 95% CI 0.622 to 0.911; *p* < 0.001, respectively) (Fig. 5a). To understand the role of antibody isotype further, we categorized samples into four groups based on low/high reactivity for NANP-specific IgG and IgM (defined by median AU) and compared C1q-fixation reactivity to CSP. Most samples were defined as either low or high for both antibody isotypes. Those with high NANP-IgG/IgM antibodies had significantly higher C1q-fixation levels to CSP (by AU = 4.9) than individuals with low NANP-IgG/IgM antibodies (*p* < 0.001) (Additional file 1: Figure S6A). Interestingly, two samples with high complement activity were classified as NANP-IgM high and NANP-IgG low, suggesting the importance of IgM for complement-fixation in these individuals. The IgG and IgM isotype reactivity of Kenyan samples to (NANP)_15_ moderately correlated with C1q-fixation to CSP (rho = 0.425, 95% CI 0.024 to 0.709; *p* = 0.034 and rho = 0.613, 95% CI 0.276 to 0.816; *p* = 0.001, respectively) (Fig. 5b) Furthermore, individuals with high NANP-IgG/IgM antibodies had only a minor increase in C1q-fixation to CSP (by AU = 1.86) than individuals with low NANP-IgG/IgM (*p* = 0.043) (Additional file 1: Figure S6B). Collectively, these data show that NANP-specific antibodies were variably associated with C1q-fixation to CSP among individuals, and between the two malaria-endemic populations.

To establish further that NANP-specific antibodies mediate C1q-fixation to CSP, we tested mouse anti-NANP MAbs (2H8 and 3C1) that had been subclass-switched so that there were 2H8-IgG1/IgG2a and 3C1-IgG1/IgG3 antibody pairs. All four MAbs demonstrated similar IgG-reactivity against CSP and (NANP)_15_, but only 2H8-IgG2a and 3C1-IgG3 effectively fixed C1q compared to their IgG1 subclass counterparts, which is known not to fix human complement (Fig. 5c) [31].

### Age-associated acquisition of naturally acquired complement-fixing antibodies to CSP

**Fig. 6:**
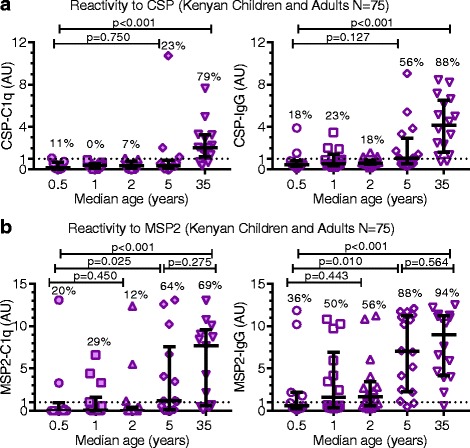
Acquisition of C1q-fixing antibodies and total IgG to CSP and MSP2 antigens in malaria-exposed Kenyan children and adults. Kenyan children and adults (*N* = 75) were categorized into groups based on the median ages 0.5, 1, 2, 5, and 35 years (*n* = 11, *n* = 14, *n* = 18, *n* = 16, and *n* = 16, respectively). Samples were tested for C1q-fixation and total IgG to CSP (**a**) and MSP2 (**b**) by ELISA. Results were standardized to arbitrary units (AU) based on malaria-naive negative controls from Melbourne (seropositivity defined as AU > 1, shown as dotted lines), and the mean of duplicates were graphed along with the group median, interquartile range, and percentage of positive samples. Reactivity between two groups and more than two groups were compared using the Mann–Whitney U test and Kruskal–Wallis test, respectively. AU arbitrary units, CSP circumsporozoite protein, ELISA enzyme-linked immunosorbent assay, IgG immunoglobulin G, MSP2 Merozoite surface protein 2

The bulk of malaria morbidity and mortality occurs in endemic areas where transmission is stable and relatively intense, particularly in children aged less than 5 years. We, therefore, investigated the acquisition of C1q-fixing antibodies to CSP among young children (*n* = 59) compared to adults (*n* = 16) living in a *P. falciparum* holoendemic area in western Kenya when transmission was high. We also compared complement-fixation responses to a major merozoite surface protein, MSP2, which is an established target of complement-fixing antibodies in blood-stage immunity [37]. In this setting, young children are known to be acquiring immunity to blood-stage infection and are susceptible to severe malaria, whereas adults have more established immunity due to extensive exposure to infections. Study participants were categorized into groups based on median ages of 0.5, 1, 2, 5, and 35 years (ages were in the ranges 0.3–0.7, 1–1.5, 1.8–3.0, 4.7–5.9, and 19.6–69.2 years, respectively). Reactivity for C1q-fixation and IgG to CSP was relatively low among children of all age groups and was significantly higher among adults (*p* < 0.001 for both tests) (Fig. 6a). In contrast, we observed higher reactivity for C1q-fixation to MSP2, a known target of naturally acquired immunity [29]. Interestingly, children in the age group with a median of 5 years had C1q-fixing antibody levels similar to adults (*p* = 0.275), which were higher than those observed in younger children (*p* < 0.001). Taken together, these results suggest that complement-fixing antibodies to CSP are poorly acquired during early childhood compared to the merozoite antigen MSP2, and that C1q-fixing antibodies to CSP are overall acquired more slowly than those to blood-stage antigens.

### High levels of C1q-fixing antibodies are associated with protection against clinical malaria in children

**Fig. 7:**
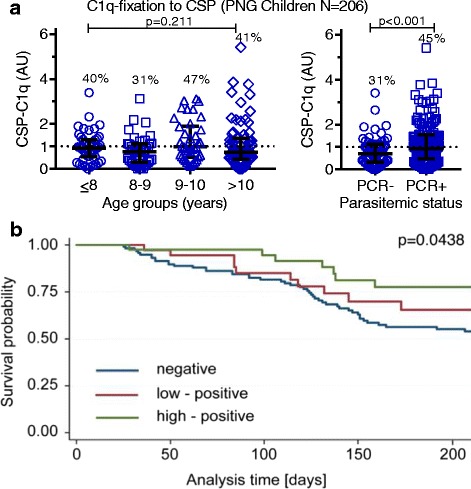
High levels of C1q-fixing antibodies to CSP are associated with protection against clinical malaria in children. PNG children (*N* = 206) were tested for C1q-fixation to CSP by ELISA. Results were standardized to arbitrary units (AU) based on malaria-naïve negative controls from Melbourne (seropositivity defined as AU > 1, shown as dotted lines). **a** C1q-fixation to CSP was categorized by age (≤8, 8.1–9, 9.1–10, and ≥10 for *n* = 47, *n* = 39, *n* = 38, and *n* = 71, respectively), and parasitemic status at enrolment by PCR. Each sample was tested in duplicate, and the mean value was used to generate box plots. The top, center, and bottom horizontal lines represent the 75th percentile, median, and 25th percentile, respectively. The upper and lower whiskers represent the highest and lowest values within the 1.5×-interquartile range, respectively. Values that exceed this range are presented as symbols. Reactivity between two groups and more than two groups was compared using the Mann–Whitney U test and Kruskal–Wallis test, respectively. **b** Kaplan–Meier survival curve showing the cumulative proportion of children who had experienced an episode of malaria during the follow-up (time in days), stratified into three groups based on C1q-fixation reactivity: negative (AU < 1), low positive (bottom 50% of positive samples, 1 < AU ≤ 1.525), and high positive (top 50% of positive samples, AU > 1.525), as shown in blue, red, and green, respectively (*n* = 116, *n* = 39, and *n* = 39, respectively; *p* = 0.0438, Wilcoxon Breslow test, unadjusted for confounders). AU arbitrary units, CSP circumsporozoite protein, ELISA enzyme-linked immunosorbent assay, PCR polymerase chain reaction, PNG Papua New Guinea

From a longitudinal cohort study of children (*N* = 206, 5–14 years) who were resident in a malaria-endemic region of PNG [25], only a minority had antibodies that promoted C1q-fixation to CSP (positivity 40%), weakly correlating with anti-CSP IgG (rho = 0.280, 95% CI 0.140 to 0.409; *p* < 0.001). When stratified into four age groups (≤8, 8.1–9, 9.1–10, and ≥10 years), there was no significant association between age and C1q-fixing antibodies (*p* = 0.211) (Fig. 7a). However, children with concurrent parasitemia at the time of enrolment as determined by the polymerase chain reaction showed significantly higher C1q-fixation activity than uninfected children (positivity 45% and 31%, respectively; *p* < 0.001) (Fig. 7a), reflecting their recent exposure to sporozoites.

To obtain epidemiologic evidence supporting the importance of antibody-mediated complement-fixation to CSP in acquired immunity to malaria, we investigated whether the minority of PNG children with high levels of functional antibodies had a reduced risk of clinical malaria. Children were treated for malaria and then actively monitored for the duration of the study, enabling us to establish any association between C1q-fixing antibody levels at enrolment and the subsequent risk of developing clinical malaria. Children were divided into three response groups based on C1q-fixation reactivity: (i) negative (AU < 1), (ii) low positive (bottom 50% positive samples, 1 < AU ≤ 1.525), and (iii) high positive (top 50% of positive samples, AU > 1.525) (Additional file 1: Figure S7). In a Kaplan–Meier survival analysis, children with high levels of C1q-fixation had a lower rate of malaria during the follow-up (Fig. 7b). Importantly, we found that children with high levels of C1q-fixing antibodies at enrolment had a significantly reduced risk of clinical malaria compared to children who were negative for C1q-fixing antibodies (Cox proportional hazards model; HR = 0.39 [0.18–0.87], *p* = 0.020), which remained significant after adjusting for potential confounders of age, location of residence, and parasitemia at baseline (adjusted HR = 0.42 [0.19–0.94], *p* = 0.034). A significant association was not observed for children with low C1q-antibodies compared to those who were negative (HR = 0.70 [0.38–1.38], *p* was not significant and adjusted HR = 0.69 [0.35–1.37], *p* = 0.2). Furthermore, there was no significant association between IgG to CSP and risk of malaria (adjusted HR 0.50 [0.19–1.32] comparing children with high IgG responses compared to IgG-negative individuals). Although the prevalence of naturally acquired functional anti-CSP antibodies was generally low, these data suggest that high levels of C1q-fixing antibodies are associated with naturally acquired anti-malarial immunity and protection against disease.

## Discussion

Developing highly efficacious vaccines requires knowledge of the functional mechanisms that contribute to protection against malaria, and the identification of correlates of protective immunity. Here we show that naturally acquired human anti-CSP antibodies can function by fixing C1q and activating the classical complement pathway, and this activity significantly correlated with IgG1, IgG3, and IgM antibodies. C1q-fixing antibodies were also shown to target the central NANP-repeat region of CSP. Importantly, we provide the first direct evidence that *P. falciparum* sporozoites are susceptible to human complement via the antibody-dependent classical pathway, resulting in enhanced traversal inhibition and sporozoite death *in vitro*. Furthermore, we found that the natural acquisition of C1q-fixing antibodies to CSP in children occurs more slowly than observed for blood-stage antigens. Although poorly acquired in general, the minority of children who acquired high levels of C1q-fixing antibodies to CSP had a significantly reduced risk of developing clinical malaria, which identified the first functional correlate of immunity to sporozoites in human studies. Together, these findings reveal that antibodies to CSP and *P. falciparum* sporozoites can function by activating human complement, and provide initial epidemiological evidence linking antibody function and protective immunity.

To detect functional anti-CSP antibodies, we measured their ability to fix C1q, which is unique to the antibody-dependent classical complement pathway, as well as downstream complement proteins. There were strong correlations between C1q fixation and C4/C2/C3/C5b-C9 fixation demonstrating that C1q-fixation alone was a sufficient measure of complement activity, which we used in subsequent cohort experiments.

We demonstrated that immune antibodies promote the deposition and activation of human complement against whole *P. falciparum* sporozoites and had functional effects. While anti-CSP antibodies can directly inhibit sporozoite traversal and invasion *in vitro* [17–19], high antibody concentrations have generally been required and the relationship between function and protection has not been established. Furthermore, the presence of anti-CSP IgG alone is an insufficient correlate of protection in humans. We found that naturally acquired human antibodies had only weak activity in the direct inhibition of *P. falciparum* sporozoite traversal, but activity was substantially enhanced by an active human complement. When using sporozoites from the NF54 line, there was a more than twofold increase in inhibitory activity when antibodies were tested in the presence of an active complement (compared to an inactivated complement), whereas substantial traversal inhibition of NF166.C8 sporozoites was seen only when antibodies were tested with active complement present. Inhibiting sporozoite traversal may be important, as it is essential for establishing liver-stage infection in animals [16, 38]. Mouse studies of the rodent species, *P. berghei*, reported that glycan-specific antibodies activated the mouse complement and inhibited the traversal and invasion of sporozoites [39]. Interestingly, this was not mediated by murine IgG2a, which we have shown is capable of fixing human C1q. There are important differences between human malaria immunity and murine models. Murine models typically use the rodent malaria *P. berghei*, which has substantial biological and functional differences from *P. falciparum*, including in the major target antigen CSP, as *P. berghei* does not have the major NANP epitope of *P. falciparum* CSP. Furthermore, complement activity in laboratory mice is much lower than for human complement, which plays an important role in immunity [40]. Murine immune responses to *Plasmodium* infection are typically dominated by murine IgG1, which does not fix complement, whereas human IgG1 and IgG3 dominate human responses and both subclasses potently activate complement. The future development of new animal models of malaria that better represent antibody–complement interactions may be valuable for vaccine development. Future research to define further the antibody titers, types, and specificity required for optimal complement fixation and functional effects would be valuable to help inform vaccine development.

Antibodies alone cannot kill target cells, but our findings suggest that they can mediate this effect by recruitment and activation of complement. We directly showed that incubation with antibodies and complement can lead to sporozoite death. It has previously been proposed that murine antibody–complement interactions lead to morphological changes and bleb formation in *P. falciparum* sporozoites [41]. However, in that prior study, complement-fixation and activation were not directly shown and the potential effect of the complement was not quantified (only qualitative data were presented), and antibodies were predominantly murine IgG1, which cannot interact with complement, as we have also shown [31]. Interestingly we found that sporozoites were partially susceptible to direct complement attack, as indicated by some direct C3 deposition and cell death. However, complement susceptibility was enhanced twofold in the presence of immune antibodies.

The NANP-repeat sequence is a known B-cell epitope of CSP, and an important target of RTS,S-induced antibodies [36]. We found this epitope also to be a target C1q-fixation using synthetic NANP peptide and NANP-specific MAbs. This supports future investigation of whether RTS,S-induced antibodies mediate complement-fixation, as they are known to predominately target this epitope. NANP-specific antibodies can neutralize sporozoite function *in vitro* [42], and can reduce liver-stage burden in humanized mice that are susceptible to *P. falciparum* [8] or mice challenged with transgenic *P. falciparum/P. berghei* sporozoites [43], although high antibody concentrations appear to be required for function. Our data suggest that complement may substantially enhance the function of these antibodies, reducing the critical concentrations needed to mediate functional effects. There were significant correlations between C1q-fixation to NANP and CSP, and between NANP-specific IgG/IgM and C1q-fixation to CSP, but the strength of correlations varied between the PNG and Kenyan populations. It is possible that antibodies to other regions of CSP mediate complement-fixation, such as epitopes in the C-terminal and N-terminal regions, which warrants further investigation.

We identified a subset of individuals from PNG and Kenya with high anti-CSP IgG reactivity that demonstrated a weak ability to fix C1q. This highlights that total IgG reactivity is not wholly reflective of functional activity, and may explain why measuring anti-CSP IgG alone is a poor correlate of protective immunity. Anti-CSP IgG can directly fix C1q, and IgM is also likely to play some role, as we observed high levels of anti-CSP IgM in selected individuals that strongly fixed C1q. Anti-CSP IgG was predominately the IgG1 and IgG3 subclasses, which are known to active complement. However, IgG2 and IgG4 were also detected in some individuals, which may negatively impact C1q-fixation by outcompeting the complement-fixing subclasses; therefore, the overall isotype and subclass profile is important for functional activity. Other antibody factors, such as epitope specificity, affinity, and glycosylation, are also important in determining function.

A striking new finding is that children with high levels of C1q-fixing antibodies to CSP had a significantly reduced risk of clinical malaria compared to children who had no detectable functional antibodies. Previously, no association between anti-CSP antibodies and protection in malaria-exposed populations has been found [44, 45]. In contrast, we show important initial epidemiologic evidence of the potential role of anti-CSP antibodies that fix complement in malaria immunity, supported by findings on the functional outcomes of antibody–complement interactions. This is the first identification of a functional antibody response to sporozoite antigens that is associated with protective immunity in malaria-exposed children, suggesting that this is a significant new correlate of immunity. Our findings suggest that although the natural development of strong complement-fixing antibodies occurs only in a minority of children, when such antibodies do develop, they contribute to protective immunity. These findings support future investigation of whether C1q-fixing antibodies are induced by vaccines such as RTS,S, and whether functional antibodies are associated with vaccine efficacy and could be used a correlate of protection.

The cell-free high-throughput assay to detect and quantify functional, complement-fixing antibodies to CSP, which we developed in this study, could be applied in large-scale clinical trials for vaccine evaluation. Although this may not wholly represent all interactions that occur on the sporozoite surface, CSP is a well-validated antigen that is known to be the major target of naturally acquired immunity to sporozoites [11, 46, 47] and is the basis of the leading vaccine candidate, RTS,S [12]. Future studies should investigate other potentially important sporozoite antigens as targets of complement-fixing antibodies and the importance of non-repeat CSP epitopes, which may have important implications in vaccine development, and whether complement activation leads to additional effects or outcomes.

## Conclusions

In summary, this study demonstrates for the first time that immune antibodies fix and activate human complement against CSP and *P. falciparum* sporozoites, which inhibited sporozoite traversal and lead to sporozoite death. These data provide key knowledge on antibody functional mechanisms against sporozoites, and help us to identify which antibody types and epitopes are important in mediating anti-malarial immunity. We also obtained promising evidence that a strong level of C1q-fixing antibodies to CSP is associated with protection against clinical malaria, and may be a valuable correlate of protective immunity. This is the first evidence of a functional antibody response to sporozoites associated with protection against malaria in children. The future application of these approaches to quantify functional immunity in vaccine trials may greatly aid our understanding of how vaccines work and why efficacy has been suboptimal in trials to date. The antibody C1q-fixation assay may be a suitable correlate of protective immunity in vaccine development and trial evaluation, and studies of acquired human immunity. Defining the functional mechanisms that confer protective immunity against malaria is essential for developing and evaluating much-needed highly efficacious malaria vaccines. These findings have strong translational significance by revealing a mechanism of anti-sporozoite immunity, and thereby enabling the development of strategies to maximize this effector mechanism in the development of highly efficacious malaria vaccines.

## Additional file

Additional file 1: Supplementary figures: Supporting data. (DOCX 1232 kb)

## Abbreviations

ABTS: 2,2′-azino-bis(3-ethylbenzothiazoline-6-sulfonic acid)
AU: Arbitrary units
CI: Confidence interval
CSP: Circumsporozoite protein
ELISA: Enzyme-linked immunosorbent assay
HIS: Heat-inactivated serum
HR: Hazard ratio
HRP: Horse radish peroxidase
IgG: Immunoglobulin G
IgM: Immunoglobulin M
MAb: Monoclonal antibody
MAC: Membrane attack complex
MSP2: Merozoite surface protein 2
NHS: Normal human serum
OD: Optical density
PBS: Phosphate-buffered saline
PI: Propidium iodide
PNG: Papua New Guinea
Rh: Tetramethylrhodamine
RT: Room temperature
RTS,S: RTS,S/AS01
SDS-PAGE: Sodium dodecyl sulphate-polyacrylamide gel electrophoresis
