# Supplementary material for: Human antibodies activate complement against Plasmodium falciparum sporozoites, and are associated with protection against malaria in children
Source: BMC Med. 2018 Apr 30;16:61. doi: 10.1186/s12916-018-1054-2 (PMC5925837; doi:10.1186/s12916-018-1054-2)
Supplement: Supplementary file 1 — Supplementary figures: Supporting data. (DOCX 1232 kb) [file 12916_2018_1054_MOESM1_ESM.docx]

**Human antibodies activate complement against *Plasmodium falciparum* sporozoites, and are associated with protection against malaria in children**

**Additional file 1: Supplementary figures**

**Authors:** Liriye Kurtovic, Marije C. Behet, Gaoqian Feng, Linda Reiling, Kiprotich Chelimo, Arlene E. Dent, Ivo Mueller, James W. Kazura, Robert W. Sauerwein, Freya J. I. Fowkes, James G. Beeson


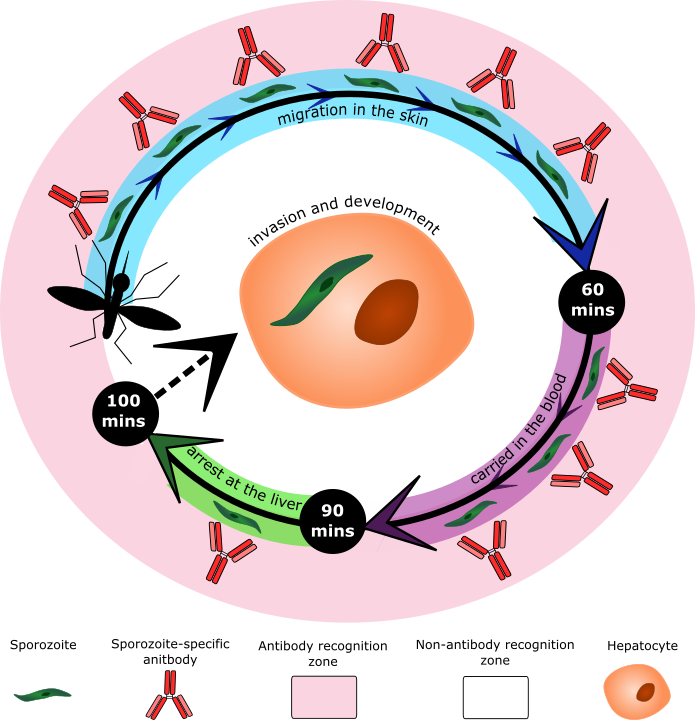


**Figure S1. Timeline of *Plasmodium* sporozoites from inoculation in the skin to establishing infection in the liver, and opportunities for immune attack.** Estimation of how long sporozoites spend in the skin and circulation before establishing liver-stage infection, where they are highly susceptible to antibody binding and complement attack. After inoculation, sporozoites spend the first 60 minutes (shown in blue) migrating in the skin using their gliding form of motility. Once sporozoites encounter a blood vessel to invade, they enter the circulatory system and are passively carried around the body (shown in purple). By 90 minutes, sporozoites are likely to arrest at the liver, where they must pass through the sinusoidal cell layer and Kupffer cells using their traversal form of motility (shown in green). Once this barrier has been breached, sporozoites are then accessible to hepatocytes to facilitate cellular invasion and development. Therefore, sporozoites are vulnerable to immune recognition for an estimated 100 minutes after inoculation, proving ample time to mount an immune response and inhibit parasite development in the liver.

**A**

**B**

HC-04 cells

+ Dextran

+ Sporozoites

HC-04 Cells

+ Dextran


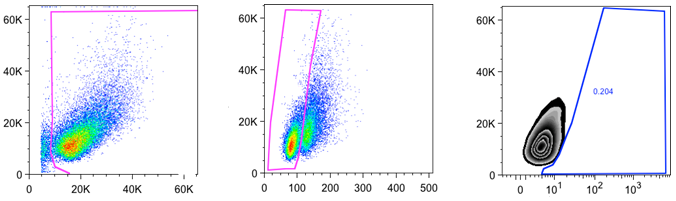

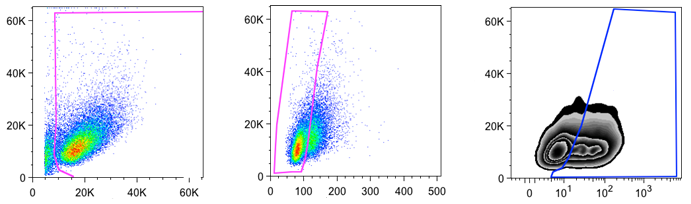


SSCSSC

FSCFSC

PW

Rh-Dextran

94.4%

59.3%

0.2%

88.5%

65%

46.5%


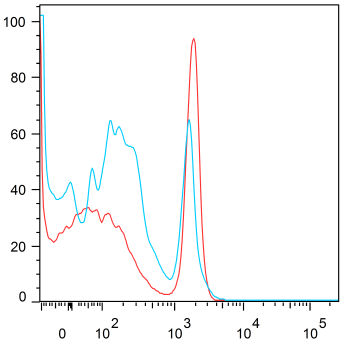


PI

% of Max

**Figure S2. Gating strategy for *P. falciparum* sporozoite traversal and sporozoite death experiments.** (**A**) Sporozoite traversal was measuring by quantifying the percentage of wounded HC-04 cells. The HC-04 cell population was gated, doublets were excluded, and wounded cells were Rhodamine-positive. Top panel: HC-04 cells incubated with dextran only (in the absence of sporozoites, antibodies and complement) were used to correct for background reactivity. Bottom panel: HC-04 cells incubated with sporozoites and dextran only (in the absence of antibodies and complement) as a positive control, showing a high proportion of Rhodamine-positive cells (i.e. wounded by sporozoite traversal). (**B**) Sporozoite death was measured by quantifying the percentage of PI-positive cells. Percentage of maximum PI-positive sporozoites treated with normal human serum and rabbit anti-CSP IgG (red line) or rabbit pre-immune IgG (blue line).


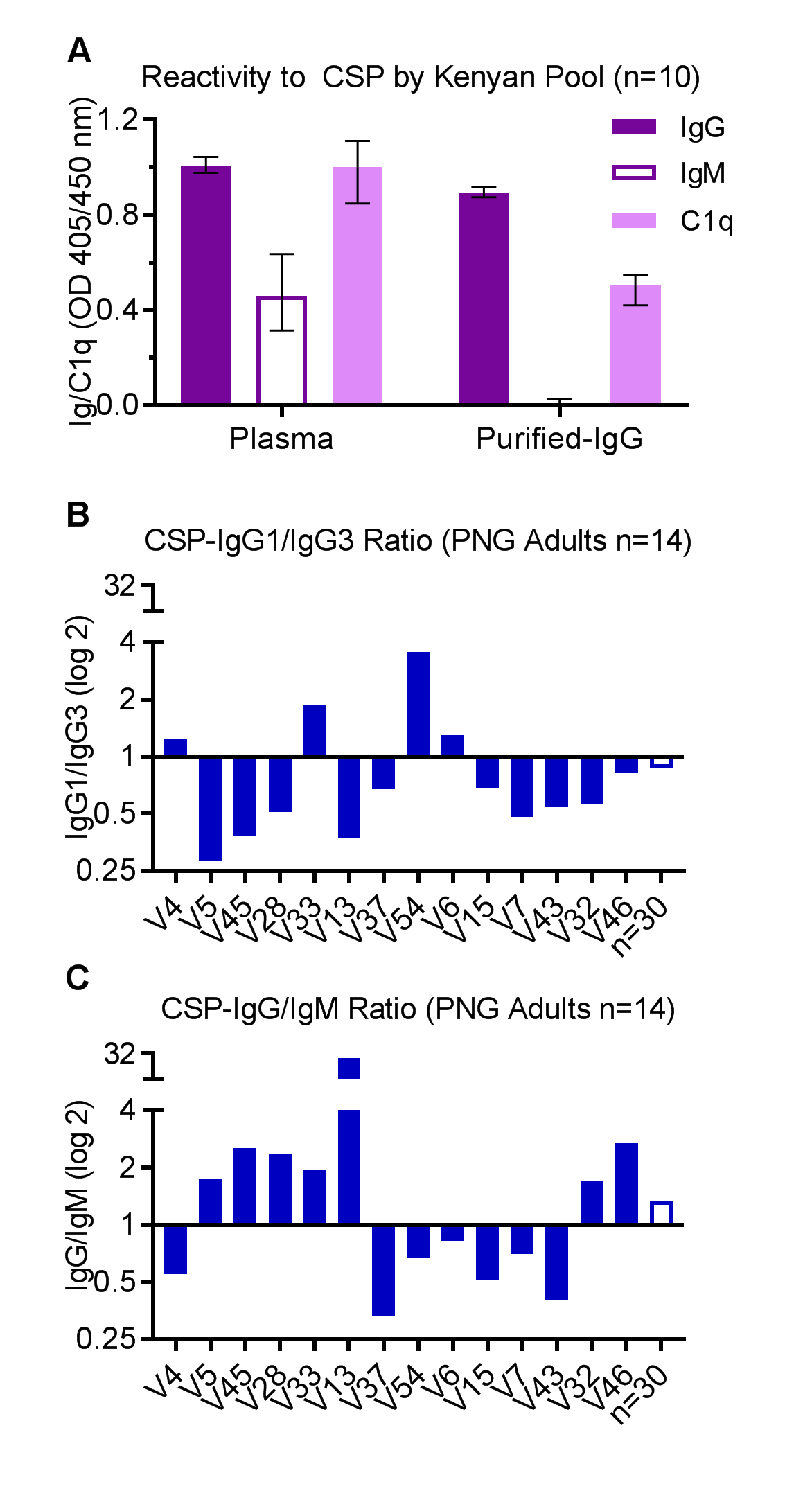


**Figure S3. The effects of antibody isotype/subclass on C1q-fixation to CSP.** (**A**) Plasma from Kenyan individuals (n=10) with high C1q-fixation reactivity were pooled, and IgG was purified (and re-constituted to the original plasma volume). The pooled plasma and purified IgG were tested for IgG, IgM and C1q-fixation reactivity to CSP, and the mean and range of duplicates were graphed. (**B-C**) Antibodies from malaria-exposed PNG adults (n=30) were tested for C1q-fixation to CSP by ELISA, and the top 50% of samples (above median AU=2.2, n=14) were examined for isotype/subclass reactivity. Samples are listed in ascending order for C1q-fixation, and the ratio of anti-CSP IgG1-to-IgG3 and anti-CSP IgG-to-IgM are shown. The group median (n=30) was also used to calculate each ratio and confirm that variability was not due to differences in assay sensitivity (open bars).

**Table S1. Linear regression to examine the relationship between antibody isotype and C1q-fixation to CSP in PNG adults (N=116).**

| **Isotype** | **Coefficient** | **Robust SE** | **95% CI** | **p-value** | **R-squared** |
| --- | --- | --- | --- | --- | --- |
| **IgG** | 0.785 | 0.101 | 0.585 to 0.985 | <0.001 | 0.67 |
| **IgG and IgM^a^** | 0.597 | 0.128 | 0.342 to 0.851 | <0.001 | 0.71 |
| **IgM** | 1.036 | 0.185 | 0.670 to 1.402 | <0.001 | 0.50 |

^a^ IgG after adjusting for IgM (variables were deemed to not be collinear , VIF=1.62). Analysis was performed in 114 individuals after the exclusion of two outliers (defines as having standardised residuals that excluded the range <-3 to >+3).


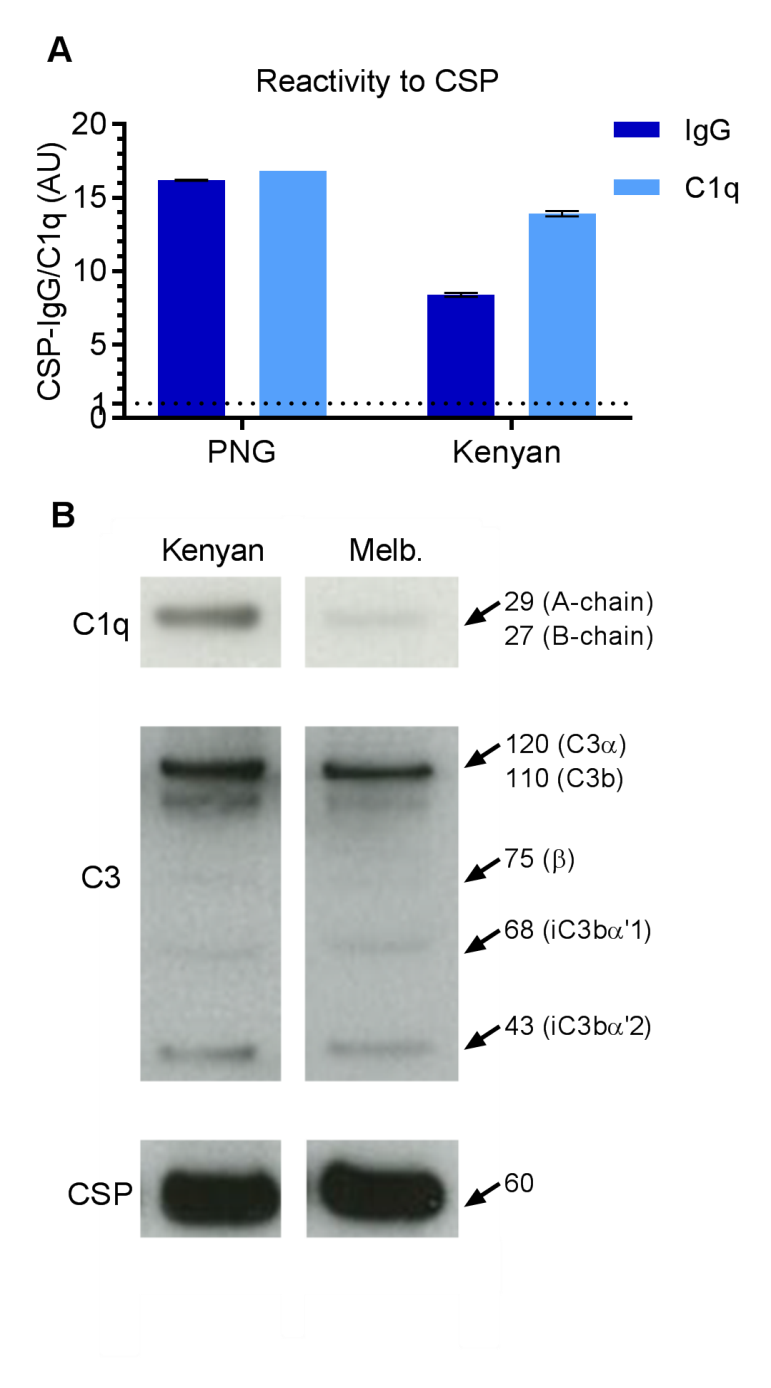


**Figure S4. Malaria-exposed individuals tested for complement-fixation to *P. falciparum* sporozoites are highly reactive to CSP, and can mediate C3b-fixation on whole sporozoites.** (**A**) IgG and C1q-fixation reactivity to CSP by malaria-exposed PNG and Kenyan individuals who were tested for complement-fixation to sporozoites (ELISA and Western blot). The results were standardized to arbitrary units based on malaria-naïve negative controls from Melbourne (seropositivity defined as AU>1), and the mean and range of duplicates were graphed. (**B**) Malaria-exposed and malaria-naïve samples from Kenya and Melbourne (respectively) were re-tested for C1q and C3-fixation to sporozoites by western blot. The predominant sporozoite surface antigen, CSP, was used as a loading control. To distinguish the alpha chain of C3 and C3b the exposure time was reduced.


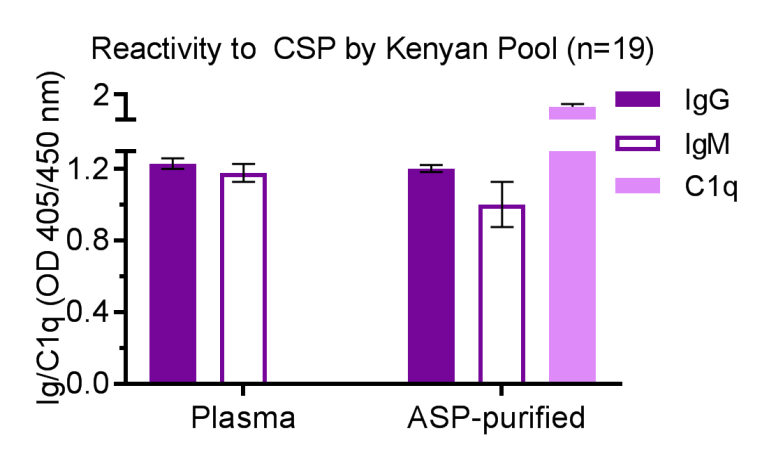


**Figure S5. Malaria-exposed Kenyan pool tested for *P. falciparum* sporozoite traversal demonstrates high reactivity for IgG, IgM and C1q-fixation to CSP.** Plasma from Kenyan individuals (n=19) with high C1q-fixation reactivity were pooled, and antibodies (including IgG and IgM) were purified by ammonium sulfate precipitation (ASP, and re-constituted to the original plasma volume). The pooled plasma and ASP-purified sample were tested for IgG and IgM-reactivity to CSP, to confirm that antibodies were not lost during the purification process. The ASP-purified sample was also tested for C1q-fixation to CSP (note that the pooled plasma was not tested), and the mean and range of duplicates were graphed.


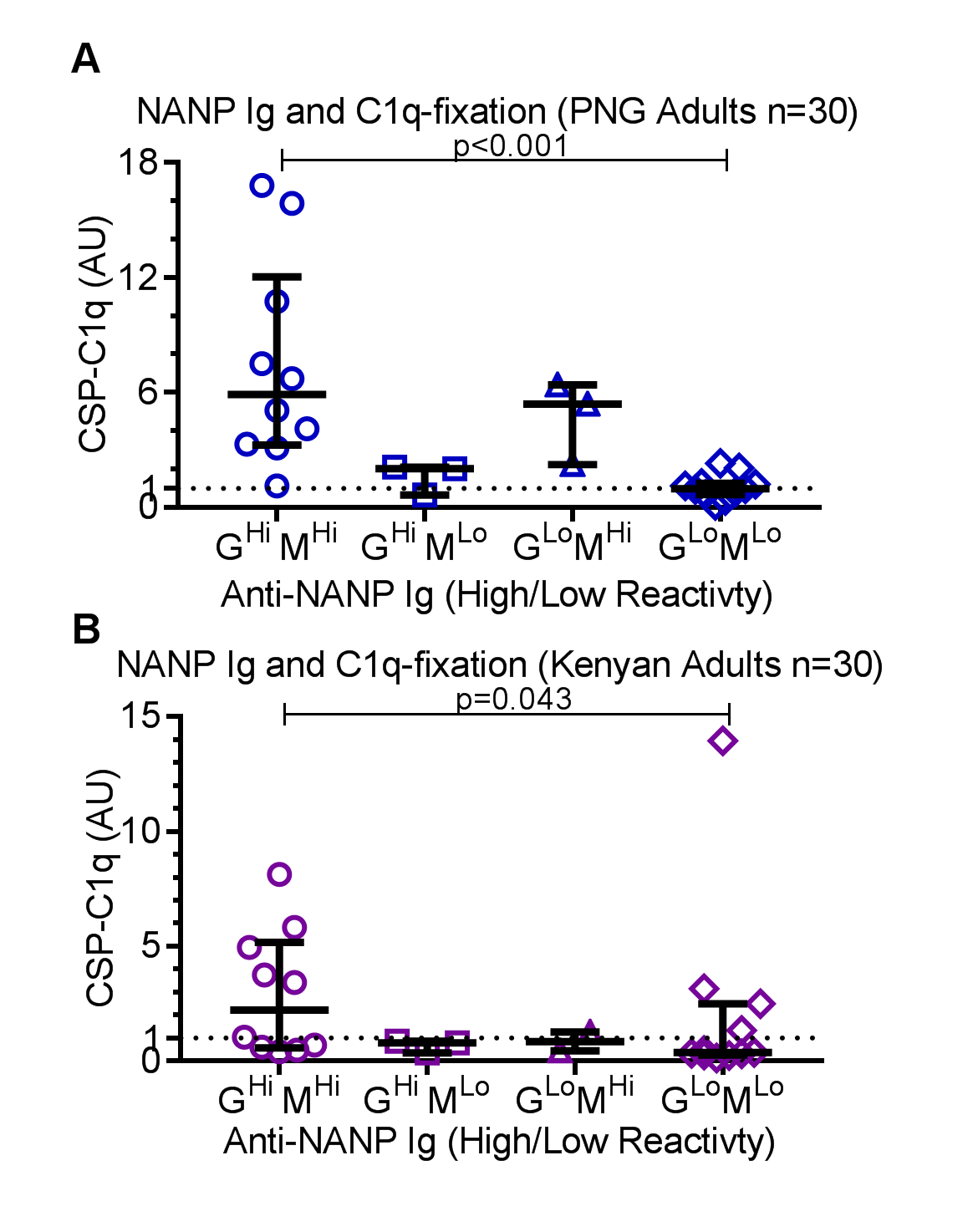


**Figure S6. NANP-specific antibodies are involved in mediating C1q-fixation to CSP.** Antibodies from malaria-exposed adults from PNG (n=30, **A**) and Kenya (n=30, **B**) were tested for IgG/IgM-reactivity to (NANP)_15_ and C1q-fixation to CSP. Samples were categorized as having high (hi) or low (lo) reactivity for anti-NANP IgG and IgM defined as above or below the median AU value (PNG: IgG=0.73 and IgM=2.18; Kenyan: IgG=1,73 and IgM=1.58). The mean of duplicates are shown, and the group median and inner-quartile range were graphed. C1q-fixation reactivity between IgG/IgM high and IgG/IgM low groups was compared using the Mann-Whitney U test.


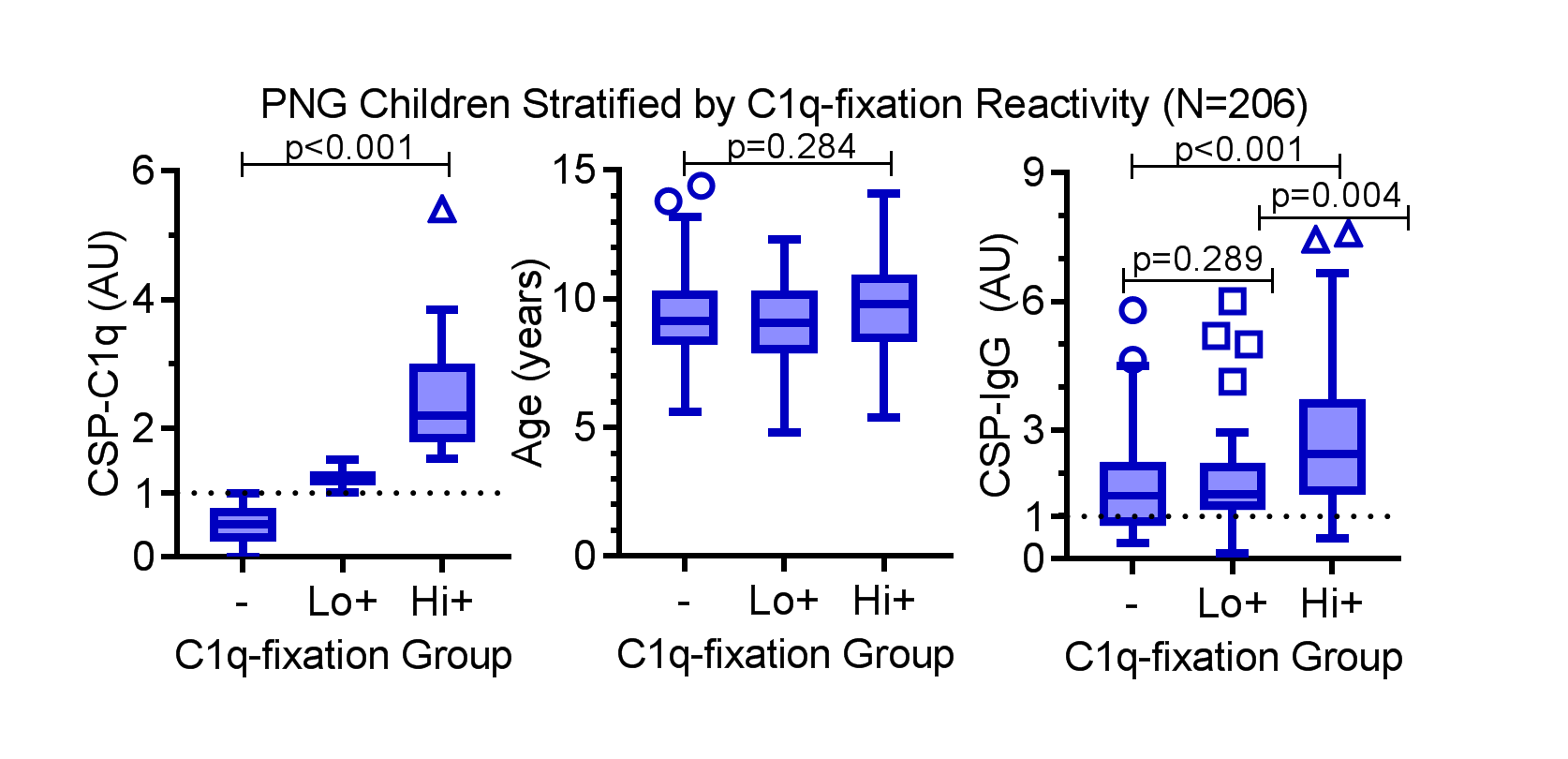


**Figure S7. C1q-fixation, age, and anti-CSP IgG among PNG children stratified by negative, low-positive and high-positive groups for C1q-fixation to CSP.** PNG children (N=206) were tested for C1q-fixation to CSP by ELISA, and categorized into three groups based on C1q-fixation reactivity; negative (-, AU<1), low-positive (lo+, 1<AU≤1.525) and high-positive (hi+, AU>1.525). C1q-fixation to CSP, age and anti-CSP IgG were categorized by C1q-fixation group. The group median, interquartile range and percentage of positive samples are also shown. Reactivity between two groups and more than two groups were compared using the Mann-Whitney U test and Kruskal-Wallis test, respectively.
